# Positional OSA part 2: retrospective cohort analysis with a new classification system (APOC)

**DOI:** 10.1007/s11325-015-1206-y

**Published:** 2015-06-18

**Authors:** M. J. L. Ravesloot, M. H. Frank, J. P. van Maanen, E. A. Verhagen, J. de Lange, N. de Vries

**Affiliations:** Department of Otolaryngology/Head Neck Surgery, Sint Lucas Andreas Ziekenhuis, Jan Tooropstraat 164, 1061 AE Amsterdam, The Netherlands; Department of Oral and Maxillofacial Surgery, Academic Medical Centre, University of Amsterdam, Amsterdam, The Netherlands; Department of Epidemiology Public and Occupational Health, EMGO Institute for Health and Care Research, VU Medical Center, Amsterdam, The Netherlands

**Keywords:** Sleep apnoea, Obstructive sleeping position, Positional therapy

## Abstract

**Background:**

In Part 1 of this two-part article, the Amsterdam Positional Obstructive Sleep Apnoea Classification (APOC) was recently introduced, a classification system aimed at facilitating the identification of suitable candidates for positional therapy (PT): patients who will benefit from a clinically significant improvement of their obstructive sleep apnoea (OSA) with PT. APOC was developed with new generation PT devices in mind rather than conventional PT (tennis ball technique). New generation PT can be defined as a well-tolerated device which prevents a patient from adopting the worst sleeping position (WSP) without negatively influencing sleep efficiency, as objectified by a full night polysomnography (PSG). PT is rapidly gaining momentum in the scope of OSA treatment. The objective of this manuscript is to measure the prevalence of position-dependent obstructive sleep apnoea (POSA) according to the APOC, in a consecutive series of patients referred for PSG as well as an investigation of associations between POSA and certain patient characteristics.

**Methods:**

We performed a retrospective, single-centre cohort study including a consecutive series of patients who underwent a PSG during the period of April 2010 until October 2010.

**Results:**

Within this OSA-cohort (*n* = 253), a prevalence of POSA of 69 % when applying APOC is measured, compared to 64 % when applying Cartwright’s classification. An inverse relation between POSA and BMI was observed, likewise between POSA and apnoea hypopnoea index (AHI).

**Conclusion:**

We are of opinion that APOC is a suitable tool to identify patients who will or will not benefit from PT, thus resulting in more cost-efficient treatment.

## Introduction

Positional therapy (PT) aims to treat patients with position-dependent obstructive sleep apnoea (POSA) by preventing patients from sleeping in the worst sleeping position (WSP) [1]. In the literature, a majority of studies apply a variation on the tennis ball technique (TBT): a bulky mass attached to the patient’s back [2]. Studies have shown that TBT is effective in reducing the apnoea hypopnoea index (AHI); nevertheless, results are unsatisfactory [2]. Ineffectiveness, backache, discomfort and no improvement in sleep quality or daytime alertness have been responsible for poor compliance rates, ranging from 40 % short-term to 10 % long-term [1, 3–5]. Recent developments have seen the introduction of a new generation of PT, a small device attached to either the neck or chest which corrects the patient from adopting the supine position through a subtle vibrating stimulus. Encouraging data have been published suggesting that this simple therapy successfully prevents patients with POSA from adopting the supine position without negatively influencing sleep efficiency, as well as allowing for good adherence both short- and long-term [6–9]. Consequently, PT, which is simple and inexpensive, shows promise as a stand-alone treatment or as an additional measure to increase the success rate of other established treatment methods [8, 10–12]. Unfortunately, evaluation of the efficacy of new generation PT and comparison of results is hindered by the fact that there are no universally used POSA criteria.

Various definitions of POSA have been applied in literature; the most common Cartwright’s criterion is a difference of 50 % or more in apnoea index between supine and non-supine positions [13, 14]. The application of various classifications hinders the comparison of the studies on PT. Furthermore, it can be questioned which classification is best suited to identify ideal candidates for new generation PT.

In Part 1 of this two-part article, the Amsterdam Positional Obstructive Sleep Apnoea (OSA) Classification (APOC) was introduced which aimed at facilitating the identification of suitable candidates for PT, specifically those patients who will benefit from a clinically significant improvement of their OSA with PT [14]. The shared use of this classification can facilitate collection of data across multiple centres and comparison of results across studies. The development and process that resulted in APOC are described in Part 1. In brief, a panel of three field experts was instructed to independently assign the diagnosis of POSA to 100 randomly selected patients; they are considered more likely to benefit from a clinically significant improvement of their OSA with PT. In a group setting, the completed lists were compared. Discrepancies were discussed until consensus was met. This resulted in the consensus standard used to calibrate the new classification. Using the nominal group technique, the APOC was developed.

**Fig. 1:**
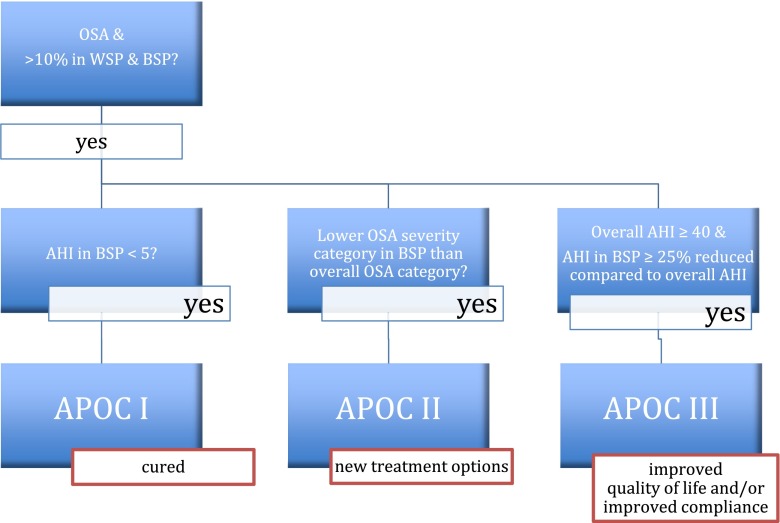
Flowchart for the Amsterdam Positional OSA Classification. The *red boxes* indicate the best possible outcome of successful positional therapy. *OSA* obstructive sleep apnoea, *AHI* apnoea hypopnea index, *WSP* worst sleeping position, *BSP* best sleeping position, *CPAP* continuous positive airway pressure, *APOC* Amsterdam Positional OSA Classification

The APOC criteria evolve around the percentage of total sleep time spent in either WSP or best sleeping position (BSP) and the AHI in BSP. On applying APOC, one discriminates between the true positional patient, the non-positional patient and the multifactorial patient, whose OSA severity is influenced in part by sleep position. The APOC defines three categories (APOC I, II, III). In clinical practise, patients meeting these criteria can be diagnosed with POSA, according to the APOC criteria (see Fig. 1).

## Material and methods

### Patients

We performed a retrospective, single-centre cohort study including a consecutive series of patients who underwent a polysomnography (PSG) during the period of April 2010 until October 2010. Patients were excluded from analysis if aged <18 years, less than 80 % sleep efficiency, failure of the position sensor and therefore unknown sleeping positions and/or in case of an AHI <5. Weight, length and date of birth were registered. The body mass index (BMI) was calculated, and the following BMI grading system was implemented: obese (BMI 30–34.9), severely obese (BMI 35–39.9), morbidly obese (BMI 40–49.9) and super obese (BMI > 50) [15]. The data described was entered in an encoded study database.

### Polysomnography

PSG recordings were carried out using a digital polygraph system (Embla A10, Broomfield, USA). This records the electroencephalogram (FP2-C4/C4-O2), electrooculogram, EKG and submental and anterior tibial electromyogram. Nasal airflow was measured by a pressure sensor and arterial oxygen saturation by finger pulse oximetry. Thoraco-abdominal motion was recorded by straps containing piezoelectric transducers. Snoring was recorded through a piezo snoring sensor. Body position was determined by a position sensor (Sleepsense, St. Charles, USA), which was attached to the midline of the upper abdominal wall. This sensor differentiated between the upright, left side, right side, prone and supine position. All signals were recorded with a digital sampling, digital filtering and digital storage (DDD) recording technology and a sample rate up to 200 Hz. Storage was done on a PCMCIA flash-card. On the following day, data were downloaded to the computer and analysed by a dedicated sleep software (Somnologica, Broomfield, USA). The data were manually reviewed for analysis by an experienced sleep investigator.

Obstructive respiratory events were analysed according to the 2007 AASM criteria [16]. Obstructive apnoeas were defined as a decrease of airflow of more than 90 % for at least 10 s, in the presence of respiratory efforts. Central apnoeas were defined as a decrease of airflow of more than 90 % for at least 10 s and no respiratory effort of the thorax or abdomen. Hypopnoeas were defined as a decrease of airflow of 30–90 % for at least 10 s, with a continuation of respiratory effort and leading to a decrease in haemoglobin saturation of at least 4 %. The AHI was calculated as the sum of total events (apnoeas and hypopnoeas) per hour of sleep. An AHI of 5–15/h is mild obstructive sleep apnoea syndrome (OSAS), an AHI of 15–30/h is moderate and AHI of >30/h is severe OSAS, as assessed by PSG.

### Statistical analysis

Statistical analysis was performed using SPSS statistical software (version 18 for osX, SPSS Inc, Chicago, USA). The distribution of recorded variables was characterised by calculating the mean and standard deviation. To satisfy assumptions, the AHI variable was transformed using the natural logarithm. The prevalence of POSA according to the traditional criteria and APOC were compared using crosstabs, chi-square test. The differences in AHI were analysed by parametric and non-parametric tests, depending on the normal distribution of the sample. The relation between POSA and patient characteristics was further evaluated employing logistic regression. A *p* value of <0.05 was considered to be statistically significant.

### POSA criteria

Both Cartwright’s criteria and the APOC were applied to the database. Patients were considered to be position-dependent according to the traditional criteria if there was a difference of 50 % or more in AHI between supine and non-supine positions. Patients were considered to be position-dependent according to the APOC if the following criteria were met:

1. OSA according to the American Academy of Sleep Medicine criteria [16],
2. >10 % of the total sleep time (TST) in both BSP and WSP,
3. a BSP AHI of less than 5,
4. a BSP AHI in a lower OSA severity category, or
5. an overall AHI of at least 40 and at least a 25 % lower BSP AHI.

## Results

### Cohort analyses

Of the 343 patients in the institutional database, who underwent a PSG during a 7-month study period, 90 patients did not meet the above mentioned inclusion criteria (Table 1).

Patient characteristics are described in Table 2. One hundred and forty-eight (63.8 %) patients met Cartwright’s criteria, and 176 (69.4 %) patients met the APOC criteria. In 56 cases (22 %), the diagnosis of POSA made on grounds of Cartwright’s classification and the APOC system did not match (*p* < 0.01). Seventeen patients were diagnosed with POSA when applying Cartwright’s criteria but did not meet the APOC criteria; all patients had mild to moderate OSA (range 8.0–29.6/h) with a relatively high WSP AHI (range 11.5–95.6/h) in combination with a high percentage non-WSP sleep time (range 40.5–89.1 %). These patients did not meet the APOC criteria as they were considered ‘self-correcting’. Thirty-nine patients met the APOC criteria but were not deemed positional when applying Cartwright’s criteria. Specifically, 14 cases had a BSP AHI <5 (APOC I); 17 cases could theoretically decrease in their OSA severity group with PT (APOC II) and eight patients with an AHI over 40 could achieve a >25 % reduction of their AHI with PT (APOC III).

**Table 1:** Best possible outcome per category of the APOC

General considerations
• OSA according to the American Academy of Sleep Medicine criteria
•>10 % of the total sleeping time (TST) in both best sleeping position (BSP) and worst sleeping position (WSP)
APOC I: patients who theoretically can be cured with PT only (resulting in an AHI <5).
• Patients diagnosed with APOC 1 if the BSP AHI <5
APOC II: patients who theoretically can decrease an OSA severity category through treatment with PT, rendering other treatment options available
• Patients diagnosed with APOC II if the AHI in the BSP falls into a lower OSA severity category than the overall AHI.
APOC III: patients with an overall AHI ≥40, who can theoretically achieve a >25 % reduction of their AHI with PT only, thereby improving compliance of existing therapies

In the second part of this two-part study, we aim to measure the prevalence of POSA according to the APOC, in a consecutive series of patients referred for PSG and its association with certain patient characteristics

*PT* positional therapy, *OSA* obstructive sleep apnoea, *AHI* apnoea hypopnoea index, *WSP* worst sleeping position, *BSP* best sleeping position, *CPAP* continues positive airway pressure, *APOC* Amsterdam Positional OSA Classification

**Table 2:** Patient characteristics: clinical and PSG parameters

	Mean (SD)	Min.–max.
Women	72 (31 %)	–
Men	160 (69 %)	–
Age (years)	50.17 ± SD 11.32	(24–81)
BMI (kg/m^2^)	31.03 ± SD 6.98	(20.7–62.4)
AHI (per hour)	25.65 ± SD 21.14	(5.0–91.0)
Mean SaO_2_ (%)	83.83 ± SD 2.34	(82.9–98)
Minimum SaO_2_ (%)	81.77 ± SD 7.88	(50–96)
DI	15.78 ± SD 18.55	(0–84)

*AHI* apnoea hypopnea index, *BMI* body mass index, *DI* desaturation index, *SaO*_*2*_ oxygen saturation, *SD* standard deviation

**Fig. 2:**
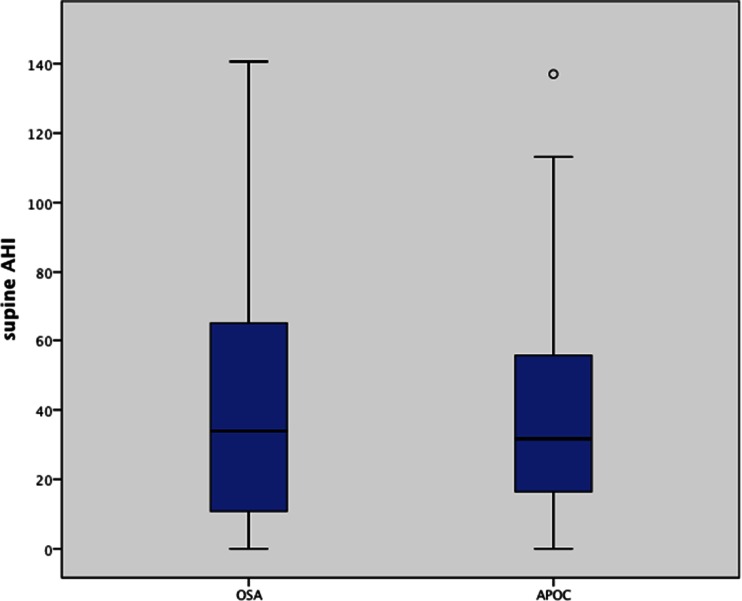
Supine AHI of patients diagnosed with OSA versus patients within APOC I–III

**Fig. 3:**
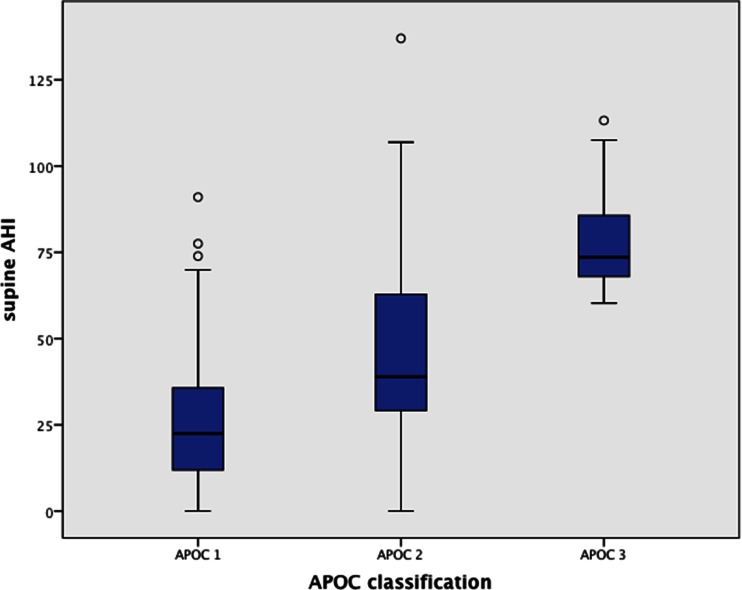
Supine AHI stratified per APOC score

Of the 176 patients who met the APOC criteria, 95 (54 %) met the APOC I criteria, 71 (40 %) APOC II and 10 (6 %) APOC III (Table 3). The most common WSP was the supine position (70.4 %). The percentage TST in the supine position was comparable in both patients with POSA (37.1 %) and OSA (35.1 %) (CI −9.08–4.96; *p* = 0.564). The supine AHI did not differ between the OSA (41.0/h) and POSA (APOC I–III) (37.4/h) group (*p* = 0.802) (Fig. 2), but within the POSA group (APOC I–III), there was a significant difference in supine AHI (*p* < 0.00) (Fig. 3). Likewise, with application of the logistic regression tests, there was no relation between the percentage of supine TST (%) and the occurrence of POSA (APOC I–III) (OR 1.003; CI 0.993–1.014; *p* = 0.563).

**Table 3:** Patient characteristics stratified per APOC category

	APOC I (*n* = 95)	*p* ^a^	APOC II (*n* = 71)	*p* ^a^	APOC III (*n* = 10)
	Count or mean (SD)		Count or mean (SD)		Count or mean (SD)
Age (years)	47.9 (11.2)	0.33	49.7 (11.0)	0.83	48.8 (11.9)
Gender M/F	67/28	0.30	44/26	0.29	8/2
BMI (kg/m^2^)	29.1 (6.1)	0.05	31.0 (5.8)	0.32	33.0 (6.5)
AHI (per hour)	12.5 (8.5)	<0.01	26.5 (11.4)	<0.01	63.8 (7.7)
Mean SaO_2_ (%)	94.5 (1.7)	0.14	94.1 (2.1)	0.01	92.9 (1.9)
Minimum SaO_2_ (%)	84.3 (10.1)	0.09	82.0 (6.0)	0.09	75.7 (10.1)
DI	5.7 (5.6)	<0.01	14.1 (10.6)	<0.01	46.2 (16.8)

*AHI* apnoea hypopnea index, *APOC*: Amsterdam Positional OSA Classification, *BMI* body mass index, *DI* desaturation index, *M* male, *F* female, *SaO*_*2*_ oxygen saturation, *SD* standard deviation

^a^ *p* is the *p* value for differences of the mean between APOC I–APOC II and APOC II–APOC III, respectively

There was an inverse relation between BMI and POSA (OR 0.949; CI 0.914–0.984; *p* < 0.01). The AHI was identified as a confounder (OR 0.956; CI 0.920–0.993; *p* = 0.02) having an inverse association to POSA itself as well (OR 0.625; CI 0.441–0.887; *p* = 0.008). This resulted in a model in which POSA was found to be inversely related to a higher BMI and AHI.

Gender was not considered to be a confounder but age was a borderline confounder (OR 0.943; CI 0.908–0.979). Males had an OR of 0.77 to develop POSA with an increase of the BMI.

When analysed per subgroup of OSA, the prevalence of POSA in the mild and moderate subgroups was similar, 68 % (Cartwright’s classification 73 %) and 81 % (Cartwright’s classification 78 %), respectively, but declined in the severe OSA subgroup, namely 57 % (Cartwright’s classification 30 %) (see Tables 4 and 5). On logistic regression analysis, moderate OSA increases the chance for POSA (OR of 2096 (CI 1.065–4.127; *p* < 0.01)) in comparison to mild OSA whilst in patients with ‘severe OSA’, the prevalence of POSA shows a non-significant decreasing trend (*B* = 0.654; CI 0.342–1.252; *p* = 0.20).

**Table 4:** Mean AHI per subgroup for Cartwright’s classification

OSA severity	Cartwright’s classification	Mean AHI (per hour)	Number (*n*)	SD (per hour)
Mild OSA	No POSA	9.0	24	2.7
	POSA	9.0	66	3.0
	Total	9.0	90	2.9
Moderate OSA	No POSA	22.5	18	4.1
	POSA	21.1	64	4.1
	Total	21.4	82	4.1
Severe OSA	No POSA	60.4	42	16.6
	POSA	47.2	18	15.9
	Total	56.4	60	17.4
Total	No POSA	37.6	84	26.1
	POSA	18.9	148	13.6
	Total	25.7	232	21.1

*AHI* apnoea hypopnea index, *OSA* obstructive sleep apnoea, *POSA* positional obstructive sleep apnoea

**Table 5:** Mean AHI per subgroup for the APOC score

OSA severity	APOC score (combined)	Mean AHI (per hour)	Number (*n*)	SD (per hour)
Mild OSA	No POSA	9.8	35	2.8
	POSA	8.7	72	2.8
	Total	9.1	107	2.8
Moderate OSA	No POSA	22.8	16	5.0
	POSA	20.7	69	3.8
	Total	21.1	85	4.1
Severe OSA	No POSA	69.2	26	15.1
	POSA	47.1	35	15.1
	Total	56.5	61	18.4
Total	No POSA	32.5	77	28.3
	POSA	21.1	176	15.8
	Total	24.6	253	21.0

*AHI* apnoea hypopnoea index, *OSA* obstructive sleep apnoea, *POSA* positional obstructive sleep apnoea

## Discussion

In this paper, we report that the prevalence of POSA according to APOC, in a consecutive series of patients referred for PSG, is different when Cartwright’s criteria are applied (69.4 % versus 63.8 %, *p* < 0.01). Having a different classification in 56 cases (22 %) translates in a significant different population identified by these classifications (*p* < 0.05). In the literature, approximately 56 % of patients with OSA are diagnosed with POSA according to Cartwright’s criteria [17–20]. In the Asian population, the prevalence has been reported to be higher at 67 % [21]. Ninety-five (54 %) met the APOC I criteria (an AHI <5). In a recent study by Teerapraipruk et al., 47 % of the patients diagnosed with POSA had a normalised respiratory disturbance index <5 in non-supine position [21].

We found that more patients were deemed positional, according to APOC, when the distribution of the AHI and selection of WSP and BSP was not only considered in supine and non-supine position but for each specific position, namely left side, right side, prone and supine position.

Similar to previous studies, we found that there are a greater percentage of patients diagnosed with POSA when applying APOC, in patients with mild OSA in comparison to patients with severe OSA (67 % versus 57 %) [17, 19, 20]. Multivariate analysis showed that the AHI was the most dominant variable that determined positional dependency, followed by the BMI [22].

In a retrospective chart review, 49.5 % of the patients with mild sleep apnoea, 19.4 % of patients with moderate sleep apnoea and 6.5 % of patients with severe sleep apnoea were found to have position-dependent OSA, defined as an overall AHI greater than 5 with a >50 % reduction in the AHI between the supine and non-supine postures and an AHI that normalises (AHI <5) in the non-supine posture [23]. And in a recent retrospective Asian study of 1170 OSA patients, positional dependency was present in 87 % of the patients with mild OSA, defined as an AHI between ≥5 and <20, in 84.2 % with moderate OSA (AHI ≥20 and <40) and in 43.1 % with severe OSA (AHI ≥40).

On further analysis of our study population, 100 % of the patients with mild OSA and diagnosed with POSA when applying APOC were classified with APOC I, whilst 25 % and 17 % of patients with moderate OSA and severe OSA, respectively, were classified with APOC III. In conclusion, the vast majority of patients with mild OSA are true positional patients, who will benefit the most from PT and could theoretically be cured by PT only. This is of clinical relevance, since patients with less severe forms of OSA are less likely to accept and thus benefit from continues positive airway pressure (CPAP) treatment, especially the less symptomatic patients [2, 24].

Even so, we advocate that more patients can benefit from PT than only true positional patients, especially since PT is simple, cheap, well tolerated and reversible. An important advantage is that the APOC discriminates between the true positional patient, the non-positional patient and the multifactorial patient, whose OSA severity is influenced in part by sleep position. We report that 75 % and 54 % of patients with moderate and severe OSA, respectively, with POSA when applying APOC, were classified with APOC II. All patients with APOC III were patients suffering from severe OSA. These patients can benefit from PT, by going down in OSA class or a decrease in AHI, resulting in less aggressive primary treatment. For example, as the AHI drops, so does the CPAP pressure needed, potentially improving tolerance and compliance.

Cartwright reported that an increase in severity of OSA in supine position relative to other positions was most striking in patients close to normal weight, whilst Oksenberg et al. found that weight changes have a modulatory effect on positional dominance and lateral AHI appears to be a sensitive parameter of these changes [2, 13, 25]. Our results confirm the inverse association between POSA and BMI [26].

### Our study has various limitations

We did not include information concerning current treatment or treatment compliance in the database. Even though patients may be diagnosed with APOC III, if they are being adequately treated with CPAP or other treatment modalities, they may not require adjuvant PT.

With the introduction of APOC, we contend that the current POSA criteria are overdue for reevaluation and open the debate to develop fitting criteria to identify patients who can clinically benefit from PT. We recognise that the combination of a continuous variable (percentage reduction of AHI) with a categorical variable (change in degree of severity of the OSA) creates unfortunate outcomes in some cases. Such shortcomings are inevitable and inherent to categorical (mixed) systems. But as we specifically seek patients who will clinically benefit from PT, we are of opinion that a patient who decreases in OSA severity category will be eligible for less cumbersome adjuvant therapy, which is a clinically relevant improvement.

Although the AHI is only a surrogate marker for OSA, it remains the most frequently reported outcome measure in OSA. Some clinicians argue that other PSG variables could be used as an outcome measure, e.g. desaturation index (DI) as a measure of intermittent hypoxia. The latter is also considered to be less susceptible to nightly variability. Others argue that clinical outcomes may be more appropriate. There are more dimensions to consider in clinical management of OSA than AHI alone, e.g. side-effects, partner acceptance or cost-effectiveness.

In retrospect, a high central apnoea index (CAHI) should have been included as an inclusion criterion. On analyses of our database, the mean CAHI was 2.45 (SD 0.43, range 0–68). Therefore, one may consider this aspect negligible. On the other hand, one can argue that one should include patients with central sleep apnoea (CSA). Despite the fact that positional dependency has not been extensively studied in CSA, available studies have shown that body position during sleep is important in about 40–50 % of patients with CSA/Cheyne Stokes breathing (CSB) [27]. Besides optimising cardiac treatment if indicated, CPAP is the first-line treatment modality in CSA/CSB. Unfortunately, therapeutic results are not always satisfactory, with an overall reduction in AHI after 3 months of about 50 %. Comparable to OSA, it is suggested that management of positional dependency is to be considered, particularly in patients with CPAP intolerance. Supporting this theory, Szollosi I et al. reported 20 patients with stable heart failure and CSA/CSB [28]. They report that the lateral body position was associated with reduced AHI-related hypoxemia during sleep stages 1 and 2. Prospective studies are needed to further explore this theory.

## Conclusion

Within this OSA-cohort, we have found a prevalence of POSA of 69.4 % when applying APOC compared to 64 % when applying Cartwright’s classification. An inverse relation between POSA and BMI was confirmed, likewise between POSA and AHI. We are of opinion that APOC is a suitable tool to identify patients who will or will not benefit from PT, thus resulting in more cost-efficient treatment. An important advantage is that the APOC discriminates between the true positional patient, the non-positional patient and the multifactorial patient, whose OSA severity is influenced in part by sleep position.

## Abbreviations

AHI: Apnoea hypopnea index
APOC: Amsterdam Positional Obstructive Sleep Apnoea Classification
BMI: Body mass index
BSP: Best sleeping position
CPAP: Continues positive airway pressure
DI: Desaturation index
OSA: Obstructive sleep apnoea
POSA: Position-dependent obstructive sleep apnoea
PSG: Polysomnography
PT: Positional therapy
SaO_2_: Saturation oxygen
SPT: Sleep position trainer
TBT: Tennis ball technique
TST: Total sleep time
WSP: Worst sleeping position
